# iPSC-Based Models to Unravel Key Pathogenetic Processes Underlying Motor Neuron Disease Development

**DOI:** 10.3390/jcm3041124

**Published:** 2014-10-17

**Authors:** Irene Faravelli, Emanuele Frattini, Agnese Ramirez, Giulia Stuppia, Monica Nizzardo, Stefania Corti

**Affiliations:** Dino Ferrari Centre, Neuroscience Section, Department of Pathophysiology and Transplantation (DEPT), University of Milan, Neurology Unit, IRCCS Foundation Ca’Granda Ospedale Maggiore Policlinico, via Francesco Sforza 35, 20122 Milan, Italy; E-Mails: iri.faravelli@gmail.com (I.F.); emanuele.frattini@gmail.com (E.F.); agnese.ramirez@gmail.com (A.R.); giulia.stuppia@studenti.unimi.it (G.S.); monica.nizzardo1@gmail.com (M.N.)

**Keywords:** induced pluripotent stem cells, amyotrophic lateral sclerosis, spinal muscular atrophy, disease modeling

## Abstract

Motor neuron diseases (MNDs) are neuromuscular disorders affecting rather exclusively upper motor neurons (UMNs) and/or lower motor neurons (LMNs). The clinical phenotype is characterized by muscular weakness and atrophy leading to paralysis and almost invariably death due to respiratory failure. Adult MNDs include sporadic and familial amyotrophic lateral sclerosis (sALS-fALS), while the most common infantile MND is represented by spinal muscular atrophy (SMA). No effective treatment is ccurrently available for MNDs, as for the vast majority of neurodegenerative disorders, and cures are limited to supportive care and symptom relief. The lack of a deep understanding of MND pathogenesis accounts for the difficulties in finding a cure, together with the scarcity of reliable *in vitro* models. Recent progresses in stem cell field, in particular in the generation of induced Pluripotent Stem Cells (iPSCs) has made possible for the first time obtaining substantial amounts of human cells to recapitulate *in vitro* some of the key pathogenetic processes underlying MNDs. In the present review, recently published studies involving the use of iPSCs to unravel aspects of ALS and SMA pathogenesis are discussed with an overview of their implications in the process of finding a cure for these still orphan disorders.

## 1. Introduction

Motor Neuron Diseases (MNDs) are incurable neurological disorders characterized by the progressive loss of pyramidal cells in the primary motor cortex (upper motor neurons, UMNs) and/or cells in the anterior horns of the spinal cord and their homologues in the motor nuclei of the brainstem (lower motor neurons, LMNs). Concerning their epidemiology, the prevalence of MNDs accounts for about 5–7 cases in every 100,000 people, with an annual incidence of approximately two new cases per 100,000 people [1]. MNDs are not equally distributed between genders, being more common in males, with a male to female ratio of 2:1 [1]. There is a great variability in life expectancy of patients affected by MNDs, for reasons that remain unknown for the most part: death usually occurs within 3–5 years after the onset of symptoms (such as for amyotrophic lateral sclerosis—ALS), but cases with either a slower (*i.e.*, spinobulbar muscular atrophy—SBMA) or a more rapid course have been described (*i.e.*, spinal muscular atrophy—SMA type 1) [2].

The biological substrate of MNDs is responsible for the extremely disabling clinical phenotype, characterized by progressive weakness with muscle wasting, eventually leading to paralysis and death, mostly secondary to respiratory insufficiency [3]. No effective therapy is currently available and the only possible treatments are limited to palliative care. Besides affecting life expectancy, MNDs highly impact on patients’ quality of life: they are usually dependent on the use of respiratory aids and wheelchairs, requiring 24-h assistance [4].

MNDs are usually differentiated relating to the subset of motor neurons that are mainly involved in the disease course. Upper motor neurons are especially affected in primary lateral sclerosis (PLS) and hereditary spastic paraplegias (HSP). On the other hand, SMA, progressive muscular atrophy (PMA), SBMA and hereditary motor neuropathies (HMNs) involve mainly lower motor neurons.

ALS is the most common adult form of MND, characterized by the simultaneous degeneration of UMNs and LMNs. ALS can be divided into a sporadic form (sALS), representing 90%–95% of all cases, and a familial form (fALS), accounting for the remaining 5%–10% of cases [5]. It is well accepted that genetic factors play a determinant role also in the sporadic ALS cases [6]. The sporadic form presents a rather uniform distribution in Western countries: in Europe and North America, the incidence is 1.5–2.7 cases per 100,000 persons every year [5], with a prevalence of 2.7–7.4 cases per 100,000 persons [7]. The incidence increases considerably every decade of life, reaching a peak at 74 years of age, and then progressively decreases [8]. The lifetime risk to develop ALS is 1:350 for males and 1:400 for females [9]. The age of onset is around 50–60 years, and the mean survival of ALS patients is 2–3 years after the diagnosis. 5%–10% of patients affected by ALS have a familial history of MNDs (fALS), in most cases presenting a mendelian autosomal dominant pattern of inheritance [6]. The clinical phenotype of fALS is usually considered indistinguishable from sALS. Nevertheless, fALS is characterized by an equal male to female ratio, a more precocious age of onset and, oftentimes, a longer life expectancy. At present, more than 16 loci have been associated with ALS or other atypical forms of MNDs, and two loci have been associated to ALS with frontotemporal dementia (ALS-FTD) [6]. In about 60% of fALS patients, a causative gene can be identified. Mutations of *C9ORF72* are found in 40% of cases, followed by mutations of superoxide dismutase-1 (*SOD1*) (20% of cases), *TARDBP* (4% of cases), *FUS* (4% of cases) and other genes [10].

The group of SMAs comprises a series of LMN disorders characterized by extreme heterogeneity in both clinical presentation and genetic condition.

The most prevalent forms of SMAs go under the name of “Proximal Spinal Muscular Atrophy”, often referred to simply as “SMA”, and are caused by genetic mutations on chromosome 5q (hence the term “5q-SMA”). SMA is the most common MND during childhood: with an incidence of 1:6000–1:10,000 live births and a carrier frequency of 1:40–1:60, it is the leading genetic cause of infantile mortality [11,12]. SMA selectively affects LMNs, being characterized by the degeneration of alpha MNs in the ventral horns of the spinal cord and MNs in the motor nuclei of cranial nerves in the brainstem. SMA patients exhibit a progressive and symmetric involvement of various muscle groups, which present hypotonic, hyposthenic and atrophic, with a preferential distribution in the proximal compartments of the lower limb. Based on the degree of severity of the disease, different forms of SMA can be identified (types I–IV). Children with type I SMA, the most severe and common form, are affected at birth or, at the latest, by the age of 6 months, thus never becoming able to sit [13]. The other types of SMA present a progressively milder phenotype. Life expectancy is extremely variable in the spectrum of the different forms of the disease, ranging from less than 2 years of age in type I, to an unaffected lifespan in type IV. The genetic condition accounting for the disease displays homozygous deletions or mutations of the survival motor neuron (*SMN*) gene mapped in 5q11.2–q13.3 [14]. In the human genome, two almost identical copies of *SMN* are localized on chromosome 5q13: the telomeric *SMN1* gene and its inverted centromeric homologue *SMN2*. *SMN2* only differs from *SMN1* for five base pair changes, of which a C to T substitution at +6 of exon 7 (c.840C > T) is the only nucleotide change in the coding region [15]. This is localized in an exonic splicing enhancer, thus causing an alternative splicing of *pre*-mRNA of *SMN2* that excludes exon 7 from the majority of *SMN2* transcripts. The result is the production of 10%–50% of the full-length functional protein and 50%–90% of a truncated, non-functional and unstable transcript (SMNΔ7) [16]. All individuals affected by SMA retain a variable number of copies of *SMN2*, which correlates to the severity of the disease. The exact functions of the SMN protein, as much as the reasons accounting for the disruption of MNs in SMA, are yet to be fully disclosed.

Further, less common forms of SMAs recognize defects in genes other than *SMN1* and present with early denervation weakness, but different clinical symptoms than those stated above, including joint contractures (infantile SMA with arthrogryposis—XL-SMA), distal rather than proximal weakness (distal SMA or HMNs), diaphragmatic paralysis (SMA with respiratory distress 1—SMARD1), and pontocerebellar degeneration (SMA with pontocerebellar hypoplasia—SMA-PCH) [17,18].

The inaccessibility to the cell type of interest majorly involved in MNDs and the lack of established models for the elucidation of pathogenetic mechanisms underlying such disorders represent a fundamental obstacle for progresses to be made in the field of therapies development. In order to resolve these issues and make durable discoveries, new strategies should be taken into account. In this respect, stem cell technology may represent a valuable solution.

**Figure 1:**
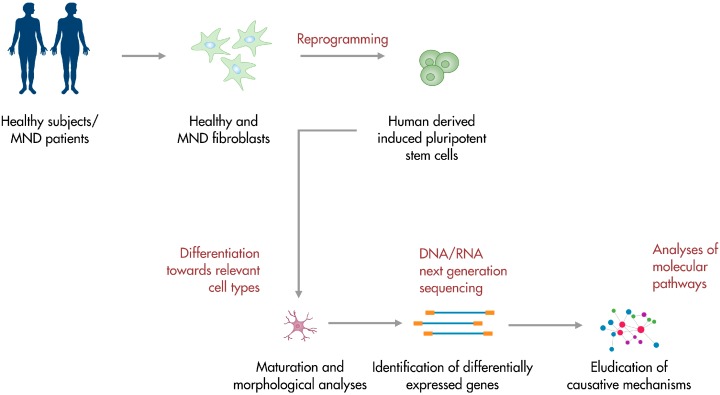
iPSC-based platforms for motor neuron disease modeling. Patients-derived somatic cells can be reprogrammed into iPSCs. Obtained cells can be differentiated towards the subtype of interest and studied in their development. Further analyses include the investigation of the transcriptional profile and the elucidation of molecular pathogenetic pathways. Human iPSCs are also a valuable tool for the identification of molecular targets and the screening of potential therapeutic compounds.

Induced pluripotent stem cells (iPSCs) are originated from patients’ differentiated cells (oftentimes fibroblasts) through the use of reprogramming factors, first identified by Yamanaka in 2006 [19]. IPSCs are able to differentiate towards cell types of the three germ layers *in vitro* and give rise to teratomas *in vivo*. They can be identified with stem cell markers and resemble embryonic stem cells (ESCs) both in morphology and behaviour, but they are not burdened by ethical concerns [20]. IPSCs are patients’ specific cells, thus avoiding more likely immunoreactions if transplantation strategies were taken into account. Moreover, iPSCs can be produced in substantial amounts, providing an optimal cell source for regenerative therapeutic approaches. The possibility of differentiating iPSCs towards any cell type of interest represents a great advantage in the context of MNDs, which affect MNs rather selectively [21]. Indeed, in recent years, protocols for the differentiation of iPSCs towards MNs have been developed and optimized, since obtaining human relevant cells appears pivotal for the development of *in vitro* models that recapitulate mechanisms responsible for the establishment of pathologies [21]. Obtained results could be crucial in guiding the process of finding an effective treatment for ALS, SMA and other MNDs. Fibroblasts can be easily obtained from skin biopsies and grown in culture, thus making the premises for a simple disease model [22]. Furthermore, MNDs with a genetic background may benefit from *in vitro* models obtained from iPSC-derived differentiated cells, like iPSC-MNs, exhibiting the affected genotype peculiar to the disease. So far, the great potential of iPSCs led to the generation of patient-specific cells for several neurodegenerative disorders, including Alzheimer’s Disease [23], Huntington’s Disease [24], Parkinson’s Disease [25] and Amyotrophic Lateral Sclerosis [26]. These considerations suggest that iPSCs could be the key to unravel pathogenetic processes behind human diseases which are challenging to study in the animal models for their specific features (Figure 1). Obtained results may pave the way to the development of effective treatments targeting specific disease mechanisms. Here, we review the recent advances in the field of iPSCs as regards their use in modeling and studying MNDs, with a focus on ALS and SMA pathogenesis.

## 2. Modeling and Studying Amyotrophic Lateral Sclerosis Using IPSCs

Amyotrophic lateral sclerosis (ALS) is a fatal neurodegenerative disease with adult onset. Symptoms reflect the dysfunction and death of motor neurons (MNs) and are characterized by muscular weakness and atrophy progressively leading to paralysis [27]. Upper and lower MNs appear to be particularly vulnerable to the disease process since they are the most relevant affected cells in the context of a relative sparing of other neuronal populations [28]. Also among MNs, specific subtypes of differently affected cells can be identified: oculomotor and Onuf’s nucleus MNs proved to be much more resistant to the disease process [29]. Reasons accounting for the selective vulnerability of MN populations and, in general, for mechanisms of neurodegeneration remain poorly understood. Several pathogenetic mechanisms have been taken into consideration, including impaired RNA metabolism, aberrant proteic misfolding, mitochondrial alterations, defective axonal transport, excitotoxicity and local inflammation [30,31]. The discovery of causative genes has given new inputs to the field. After the first report of ALS causative mutations in the gene encoding the Cu/Zn-dependent antioxidant enzyme superoxide dismutase-1 (*SOD1*) [32], researchers have started to investigate non cell-autonomous mechanisms linked to the development of ALS disease (*i.e.*, the role of the oxidative damage). The identification of *C9ORF72* repeat expansion as the major factor responsible for ALS onset in the familial forms has focused attention on the causative role of alterations in RNA metabolism [33,34,35], a line of research supported also by the involvement of mutations in *TARDBP* and *FUS* genes (encoding DNA/RNA binding proteins) in ALS development [36].

The establishment of human cell platforms has allowed for the first time to test *in vitro* some of these pathogenetic hypotheses and to model and investigate early disease mechanisms. Eggan’s group pioneered the field in 2008, when they investigated the potential of human MNs derived from embryonic stem cells (ESCs) to provide key data on ALS pathogenesis [37]. ESC-derived human MNs were cultured on primary cortical glia obtained from *SOD1* mutated mice. After 10 days in culture, a significant decrease in MN number could be observed in a time-dependent manner, thus suggesting a strong implication of non-cell autonomous mechanisms in ALS onset. Moreover, they tested if the same toxic glial effect could be detected with human interneurons and they found that the glia-induced toxicity is rather specific for MNs: interneurons treated for 20 days with *SOD1G93A* glia-conditioned medium appeared fully preserved. Complementary, MNs co-cultured with *SOD1G93A* mouse embryonic fibroblasts remained unaffected, thus indicating that the toxic effect is specifically related to the presence of astrocytes. Oligonucleotide arrays were exploited to identify genes differentially expressed in mutant glia. After these analyses, Di Giorgio and colleagues focused their investigation on the role of prostaglandin D2, which resulted responsible for a significant decrease of MN survival in culture. This work proved the advantages to use human cells for *in vitro* disease studies and provided the basis for further investigations on ALS pathogenesis.

The same research group proceeded beyond these results with a recent study aiming to elucidate pathways which are impaired by the expression of mutated *SOD1* in human MNs [26]. Kiskinis and colleagues derived iPSCs from skin fibroblasts of ALS patients; these cells harbored the patient-specific genetic combination, thus providing a precious tool to model the development of human pathology. Human iPSCs were differentiated towards MNs and compared with two healthy human iPSC lines. Diseased MN number decreased in culture in a time-dependent manner, a process that did not affect the non-motor neuronal cells present in the plate. Both control and *SOD1* iPSC lines presented a further reduced survival when co-cultured with *SOD1* glia, but this effect was much more amplified in the latter case implying the presence of a strong cell-autonomous component in ALS pathology. *SOD1* MNs presented also an unhealthy morphology with shorter processes and reduced soma, thus summarizing the changes observed in the human pathology. Importantly, ZFN-mediated gene correction of *SOD1* mutation resulted in the rescue of both altered morphology and reduced lifespan. Data from RNA-seq of *SOD1* MNs highlighted a strong down-regulation of genes related to mitochondria functions and protein translation. Further analyses showed impairment in mitochondria motility in addition to an enrichment of mitochondria number located in neuronal processes. A substrate of ER stress has been found in healthy human MNs, which could be related to the cell size; this result is in line with the well-known early degeneration of largest alpha MNs in ALS. A combination of oxidative and ER stress and the up-regulation of the unfolded protein response (UPR) have been shown and could be additive causative mechanisms of neuronal toxicity. Moreover, the electrical activity of MNs could be involved in the ER stress, a result which nicely correlates with the report of the intrinsic hyperexcitability displayed by iPSCs-derived MNs from ALS patients [38]. Finally, comparison between human-iPSCs derived *C9ORF72* and *SOD1* lines led to the discovery of common pathways downstream of these mutations related to enhanced oxidative stress response and decreased mitochondria activity.

*C9ORF72* iPSC lines were obtained by Sareen *et al.* to investigate the pathological processes underlying the most common genetic form of ALS [39]. They investigated whether the toxicity linked to the repeat expansion in *C9ORF72* was due to either gain of function or loss of function or both mechanisms. To address this question, iPSCs lines were developed from different *C9ORF72* hexanucleotide expansion carriers affected by ALS and/or frontotemporal lobar degeneration (FTLD). Southern blot analyses were performed on derived MNs assessing the expansion and also highlighting differences among patients, which in some cases could be reflected in more severe forms of the disease. FISH analyses detected the presence of RNA foci within *C9ORF72-*ALS MNs (and also in the neuronal progenitors and astrocytes), in line with previous data from patients’ tissues [40]. Observed RNA foci co-localized with Pur-alpha and hnRNAP1 but not with FUS or TDP43, responsible for other genetic forms of ALS. However, hnRNAP1 is known to interact with TDP43 [41] and its involvement in the disease process could suggest an indirect connection between *C9ORF72* and TDP43 ALS forms. This work provided data in the direction of a “gain of function” mechanism linked to *C9ORF72* hexanucleotide expansion. It has been shown that the mutated allele is usually transcribed and its downregulation using antisense oligonucleotides did not affect cell viability, but resulted in the correction of cell transcriptional profile. The specific pathogenetic role of the involvement of different RNA binding proteins interacting with RNA foci needs further investigations and could represent a common causative mechanism shared by different forms of ALS. Indeed, the aberrant cytoplasmatic aggregation of TDP43 represents a rather common pathological hallmark both in familial and sporadic ALS. Processes leading from the cytoplasmic aggregation to the selective MN loss appear to be exquisitely human and not easily detectable in animal models, where overexpression of TDP43 is ubiquitously provoked. Indeed, the majority of cell platforms and animal models considered in ALS pathogenesis studies relied on the overexpression of TDP43 in nonhuman or nonneuronal cells. Reason underlying the selective vulnerability of MNs to the disease process could be misled as well as the investigation of key molecular events that cause the human disease [40]. The establishment of human iPSC-derived platforms has allowed significant advances in the field. Bilican *et al.* generated iPSCs from a patient affected by ALS carrying the TDP43 M337V mutation and used them as a tool to investigate TDP43 pathology in human neuronal cells [42]. No differences in the differentiation and maturation towards a motor neuronal fate could be observed between TDP43-iPSCs and healthy controls. However, affected MNs presented reduced survival in culture and higher levels of soluble and detergent-resistant TDP43, probably due to an alteration in post-translational mechanisms. Interestingly, mutant MNs appeared to be vulnerable to PI3K inhibition, while they were not affected by inhibitors of other kinase pathways. This suggests also a specificity in the neuronal response to different neurotrophic factors involved in PI3K rather than MAPK pathways. The role of neurotrophines in the survival of TDP43 MNs is worthy of further investigation and this work opened up the path to the use of TDP43-ALS patients’ iPSCs as a valid disease model.

Alami *et al.* proceeded beyond these data to elucidate the role of TDP43 in physiological conditions and its impairment in ALS related disease [43]. Using at first a Drosophila model, they discovered that TDP43 cytoplasmatic granules are motile and dynamically transported along axons. Further studies in murine cortical neurons showed that this transport is microtubule-dependent and resulted in impaired in TPD43 mutated neurons, where granules appeared more immotile and often reversed direction. Other forms of axonal transport, such as mitochondria movement, were unaffected, thus suggesting a selective function and, consequently, alteration of TDP43 granules. These granules were found to be directly involved in the transport of specific mRNAs, such as Neurofilament-L (NEFL) mRNA, along the axons. To avoid influences related to the overexpression of TDP43 in the animal models, these results needed to be validated in patients’ iPSC-derived MNs. NEFL transport was analyzed in iPSC-MNs derived from patients carrying the same mutations studied in Drosophila and murine neurons (M337V and A315T) plus G298S. An impairment of the anterograde transport of NEFL granules was demonstrated together with an increase of retrograde movement. It is also important to highlight that TDP43 domain affected by the mutation is a prion-like domain known to be involved in the assembly of RNA granules. This study provided important data on a physiological function of TDP43 cytoplasmatic granules and how their impairment could significantly contribute to ALS pathology.

Neurofilament aggregation is a well-known pathological hallmark of ALS. Thanks to iPSC-based technology, Chen *et al.* could investigate the causative role of mutant *SOD1* in impairing neurofilament (NF) turnover within MNs [44]. To bypass concerns related to heterogeneity among individuals, they generated ALS patients’ iPSCs and used transcription activator-like effector nucleases (TALEN) technology to correct the *D90ASOD1* mutation, thus obtaining isogenic controls. A modified differentiation protocol was applied to originate synchronized mature cells avoiding the generation of later-born MNs or glial cells.

Indeed, challenges that need to be overcome when producing iPSC-derived neurons involve both the generation of immature cells and significant differences in the time rate of differentiation among iPSC colonies. These issues could lead to the production of heterogonous populations in terms of neural differentiated phenotype. The optimization of differentiation protocols has allowed generating neural cell populations that are synchronized regarding their growth in culture, in order to properly observe and investigate all the phases of their development [42].

NF subunits in ALS-MNs appeared to be unbalanced and developed a tendency to aggregate leading to neurite degeneration, an effect that probably interests the early phases of the disease *in vivo* when patients present phenotypically asymptomatic or with very mild symptoms. This effect was due to the presence of mutated *SOD1*, as demonstrated by gain of function and loss of function experiments. Further analyses revealed that binding of *SOD1* to 3′ UTR of NF-L mRNA could cause the alteration of NF structure, making them prone to aggregate. These events were selectively present in ALS-MNs and not in control cells or non-MNs. Moreover, human ALS MNs presented *in vitro* lower/normal levels of *SOD1* (and increased amount of NF) compared to control MNs, strongly contrasting data from animal models where mutant *SOD1* levels are much higher.

Overall, these results highlight the importance of validating data obtained from animal models in human cells and using them as a precious tool to elucidate mechanisms which are peculiar of human physiology and pathology. The identification of the mechanisms underlying the development of ALS is crucial for the discovery of new therapeutic compounds. Indeed, the elucidation of the role of misregulated neurofilament turnover [44], granular impaired trafficking [43] rather than the mitochondrial dysfunction [26], together with other recent discoveries [45], may be a key moment in the identification of specific molecular targets for the development of effective therapies. Most likely, an effective therapeutic approach should be as comprehensive as possible to counteract the multiple aspects of ALS multifactorial pathogenesis. Certainly, the use of human cells for disease modeling *in vitro* has provided crucial data for this purpose (Table 1). The optimization of iPSC-based platforms can also represent an effective tool for *in vitro* screening of potential therapeutic compounds previously identified [46]. Finally, it is worth mentioning that Kondo *et al.* reported very recently how local transplants of human iPSC-derived glial-rich neural progenitors (hiPSC-GRNPs) were able to reduce MN degeneration and increase lifespan in a murine model of mutant *SOD1* ALS [47]. ALS astrocytes are known to contribute to neuroinflammation and neuronal death within the spinal cord. HiPSC-GRNPs represent a renewable source of autologous cells able to give rise to healthy astrocytes, which can provide trophic support to endogenous diseased MNs. Kondo and colleagues observed an improvement of ALS pathology even when hiPSC-GRNPs were transplanted after the disease onset (the most probable clinical setting) thus providing evidence that glia cells could play a major role in ALS pathogenesis and, eventually, therapy.

**Table 1:** iPSC-Based studies on amyotrophic lateral sclerosis/spinal muscular atrophy (ALS/SMA) pathogenesis.

Reference	Cells	Reprogramming Method	Differentiation Protocol	Mechanism
Di Giorgio *et al.* 2008 [37]	Human ESC-derived MNs	-	Human ESC media without FGF2 or plasmanate + RA (Sigma) (1 μM) and an agonist of the SHH signaling pathway (1 μM) in N2 media: 1:1 DMEM:F12 + Glutamate (Gibco), penicillin (10,000 units) and streptomycin (Gibco) (1 mg/mL), N2 Supplement (Gibco) (1%), AA (Sigma-Aldrich) (0.2 mM), d-(+)-Glucose (Sigma-Aldrich) (0.16%), BDNF (R&D) (10 ng/mL), for 14 days.	MNs co-cultured with *SOD1G93A* astrocytes undergo cell death.
				Prostaglandin D2 is responsible for the decrease in MN survival.
Kiskinis *et al.* 2014 [26]	Fibroblasts from *SOD1A4V* ALS patients → iPSC-derived MNs	Retroviral transduction (KLF4, SOX2, OCT4, and c-MYC)	DMEM/F12, KSR (15%) on days 1–4;	*SOD1* iPSCs and MNs suffer a reduction in survival when co-cultured with *SOD1* glia. *SOD1* MNs exhibit shorter processes and reduced soma. Gene correction of *SOD1* mutation rescues both morphology and survival.
			DMEM/F12 with l-glutamine, NEAAs, HE (2 μg/mL), N2 supplement (Gibco) on days 5–24;	*SOD1* MNs show a down-regulation of genes implied in mitochondria homeostasis.
			SB431542 (Sigma) (10 μM) + DM (Segment) (1 μM) on days 1–6;	Oxidative and ER stress and the up-regulation of UPR may contribute to neuronal toxicity.
			BDNF (R&D) (10 ng/mL), AA (Sigma) (0.4 mg/mL), RA (Sigma) (1 μM) and SAG 1.3 (Calbiochem) (1 μM) on days 5–24.	*C9ORF72* and *SOD1* MNs share common disrupted pathways leading to enhanced oxidative stress response and decreased mitochondria activity.
Sareen *et al.* 2013 [39]	Fibroblasts from *C9ORF72* ALS patients → iPSC-derived MNs	Episomal plasmid nucleofection (OCT4, SOX2, KLF4, L-MYC, LIN28, and p53 shRNA)	IMDM supplemented with B27-vitamin A (2%) and N2 (1%) on days 1–6;	RNA foci in *C9ORF72* MNs co-localize with hnRNAP1, suggesting an indirect connection between *C9ORF72* and TDP43 ALS forms.
			addition of all-trans RA (0.1 μM) on days 6–25;	The toxicity linked to *C9ORF72* hexanucleotide expansion may be due to a “gain of function” mechanism.
			Neurobasal, B27 (2%) and N2 (1%) + RA (0.1 μM) and PMN (1 μM) on days 17–25;	
			DMEM/F12, B27 (2%), RA (0.1 μM), PMN (1 μM), db-cAMP (1 μM), AA (200 ng/mL), BDNF (10 ng/mL), and GDNF (10 ng/mL) for a further 2–7 weeks;	The downregulation of the mutated allele with antisense oligonucleotides corrects the cell transcriptional profile.
Bilican *et al.* 2012 [42]	Fibroblasts from TDP43 M337V ALS patients → iPSC-derived MNs	Retroviral transduction (KLF4, SOX2, OCT4, and c-MYC)	Chemically defined medium supplemented with SB431542 (Tocris) (10 μM), DM (Calbiochem) (2.5 μM), and NAC (Sigma) (0.5 μM) for 5–7 days;	TDP43 MNs present a reduced survival in culture and high levels of TDP43 due to aberrant post-translational mechanisms.
			chemically defined medium with RA (Sigma) (0.1 μM) for 7–12 days;	
			Neurobasal medium (Invitrogen), RA (0.1 μM), PMN (Calbiochem) (1 μM), N2 supplement (Invitrogen) (1%), NEAAs (Invitrogen) (1%), penicillin/streptomycin (Invitrogen) (1%), GlutaMAX (Invitrogen) (1%), and basic FGF (5 ng/mL) for 7–10 days;	Neuronal response to neurotrophic factors involved in PI3K pathways influences TDP43 MN survival.
			Neurobasal medium (Invitrogen), N2 supplement (Invitrogen) (0.5%), NEAAs (Invitrogen) (1%), penicillin/streptomycin (Invitrogen) (1%), GlutaMAX (Invitrogen) (0.5%), BDNF (PeproTech) (10 ng/mL), GDNF (PeproTech) (10 ng/mL), and F (Tocris) (10 μM) for 3–6 weeks.	
Alami *et al.* 2014 [43]	Fibroblasts from TDP43 A315T and TPD43 G298S ALS patients → iPSC-derived MNs	Retroviral transduction (OCT4, SOX2, and KLF4)	KSR medium (KO-DMEM (Life Technologies) supplemented with KSR (Life Technologies) (15%), 1 × Gibco GlutaMAX (Life Technologies) and NEAAs (100 μM) on days 0–10;	The microtubule-dependent transport of NEFL mRNA granules along the axon is impaired in TDP43 MNs.
			N2 medium (Neurobasal (Life Technologies)) supplemented with 1 × N2 (Life Technologies), 1X Gibco GlutaMAX (Life Technologies) and NEAAs (100 μM)) on days 4–14;	
			SB431542 (Sigma) (10 μM) and LDN-193189 (Segment) (100 nM) on days 0–5;	TDP43 domain affected by the mutation is involved in the assembly of RNA granules.
			RA (Sigma) (1 μM), SAG (EMD Millipore) (1 μM), DAPT (EMD Millipore) (5 μM) and SU-5402 (Biovision) (4 μM) on days 2–14;	
			murine glia-conditioned N2 medium supplemented with 1 × B-27 (Life Technologies), and BDNF (10 ng/mL), GDNF (10 ng/mL) and CNTF (R&D) (10 ng/mL).	
Chen *et al.* 2014 [44]	Fibroblasts from *SOD1A4V* and *SOD1D90A* ALS patients → iPSC-derived MNs	Non-integrating Sendai virus transduction (OCT3/4, SOX2, KLF4, and c-MYC)	DMEM/F12, N2 supplement, NEAAs, SB431542 (2 μM), LDN193189 (300 nM), and CHIR99021 (3 μM, all from Stemgent, Cambridge, MA, USA) on days 1–7;	Binding of *SOD1* to 3′ UTR of NF-L mRNA may be responsible for neurofilament tendency to aggregate leading to neurite degeneration.
			addition of RA (0.1 μM) and PMN (0.5 μM) on days 8–14;	Unlike mice *SOD1* MNs, human *SOD1* MNs have lower or normal levels of *SOD1* compared to control MNs.
			DMEM/F12, N2 supplement, and NEAAs on days 14–21.	
Ebert *et al.* 2009 [48]	Fibroblasts from a *SMN1* SMA type I patient → iPSC-derived MNs	Lentiviral transduction (OCT4, SOX2, NANOG, and LIN28)	NIM (1:1 DMEM/F12 and N2 supplement (Gibco) (1%)) supplemented with RA (0.1 μM) for 1 week;	SMA iPSCs show reduced levels of SMN full-length transcripts, due to *SMN1* loss, and a few truncated transcripts lacking exon 7.
			addition of SHH (R&D) (100 ng/mL) for 1 week;	After a robust production, SMA iPSC-derived MNs undergo a reduction in number and size compared to WT iPSC-derived MNs.
			RA and SHH medium supplemented with cAMP (1 mM), AA (200 ng/mL), BDNF and GDNF (both 10 ng/mL, PeproTech Inc., Rocky Hill, USA) for 2–6 weeks.	MN ontogenesis in SMA is disrupted by post-development damage.
Sareen *et al.* 2012 [49]	Fibroblasts from a *SMN1* SMA type I patient → iPSC-derived MNs	Episomal plasmid nucleofection (OCT4, SOX2, NANOG, and LIN28)	Stemlin Neural Expansion Media (Sigma) supplemented with EGF (100 g/mL), FGF-2 (100 ng/mL), and HE (5 μg/mL) for 3 weeks;	SMA MNs show increased levels of cleaved caspase-3 and caspase-8 and membrane-bound Fas ligand, suggesting that apoptosis is implied in MN dysfunction and loss in SMA.
			NIM (1:1 DMEM/F12 and N2 (1%)) in the presence of all-trans RA (0.1 μM) for 1 week;	The administration of Anti Fas-Ab rescues MN survival in *in vitro* models of SMA.
			addition of PMN (1 μM) or SHH (10 ng/mL) for 1–3 weeks.	
Corti *et al.* 2012 [20]	Fibroblasts from *SMN1* SMA type I patients → iPSC-derived MNs	Episomal plasmid nucleofection (OCT4, SOX2, NANOG, LIN28, c-MYC, and KLF4)	DMEM/F12 (Gibco, Invitrogen), supplemented with MEM NEAAs solution, N2, and HE (Sigma-Aldrich) (2 mg/mL) for 10 days;	SMA MNs show a reduction in size, axonal elongation, neuromuscular junction production and overall decreased survival.
			addition of RA (Sigma-Aldrich) (0.1 μM) for 7 days;	SMA MNs exhibit a different splicing profile in a subset of genes encoding proteins involved in RNA metabolism, MN differentiation, axonal guidance and signal transduction.
			same medium with RA (0.1 μM) and SHH (R&D) (100–200 ng/mL) for 7 days;	Gene correction of *SMN2* with antisense oligodeoxynucleotides rescues the cellular damage and the altered splicing profile secondary to *SMN1* deficiency *in vitro*.
			addition of BDNF, GDNF, and IGF-1 (PeproTech) (10 ng/mL) on day 24.	
McGivern *et al.* 2013 [50]	Fibroblasts from *SMN1* SMA patients → iPSC-derived glia	Lentiviral transduction (OCT4, SOX2, NANOG, and LIN28); Episomal plasmid nucleofection (OCT4, SOX2, NANOG, LIN28)	Human neural progenitor growth medium (Stemline, Sigma-Aldrich) supplemented with basic FGF-2 (Chemicon) (100 ng/mL), EGF (Chemicon) (100 ng/mL), and HE (Sigma-Aldrich) (5 μg/mL);	SMA astrocytes show increased basal calcium levels with a minimal response to ATP and an activated state that precedes MN loss.
			DMEM: Nutrient Mixture F12 (Invitrogen) supplemented with B27 (Invitrogen) (2%) with or without CNTF for 2–8 weeks.	The ERK apoptosis pathways of SMA MNs may be initiated by the defective calcium homeostasis and the deficiency of trophic factors.

AA = Ascorbic Acid; BDNF = Brain-Derived Neurotrophic Factor; cAMP = cyclic Adenosine MonoPhosphate; CNTF = Ciliary Neurotrophic Factor; DAPT = Difluorophenacetyl-Alanyl-Phenylglycine-T-butyl ester; DM = Dorsomorphin; DMEM = Dulbecco’s Modified Eagle Medium; EGF = Epidermal Growth Factor; F = Forskolin; FGF-2 = Fibroblasts Growth Factor-2; GDNF = Glial cell line-Derived Neurotrophic Factor; HE = Heparin; IGF-1 = Insulin-like Growth Factor-1; IMDM = Iscove’s Modified Dulbecco’s Medium; KSR = Knock-out Serum Replacement; NAC = *N*-Acetyl-Cysteine; NEAAs = Non-Essential Amino Acids; NIM = Neural Induction Medium; PMN = Purmorphamine; RA = Retinoic Acid; SAG = Smoothened Agonist; SHH = Sonic Hedgehog.

## 3. Modeling and Studying Spinal Muscular Atrophy Using IPSCs

Ebert and Svendsen were the first authors to demonstrate that human iPSCs can be employed to recapitulate the specific pathology that underlies SMA [48]. Until then, the only models available for research purposes on the disease were provided by worms, flies and mice, which presented considerable limitations, including technical constraints like the necessity of performing complicated knockout strategies. Similarly, human fibroblasts did not prove to be suitable for the study of the disease mechanisms and the screening of new drug compounds, since the SMN protein presents features that are peculiar to neural line-belonging cells. Applying lentiviral infection methods, SMA iPSCs were generated from fibroblasts of a 3 years old boy affected by type I SMA. Wild-type (WT) iPSCs derived from fibroblasts of his unaffected mother served as controls. Through RT-PCR analysis, that was performed in both WT and SMA iPSCs and fibroblasts in order to assess SMN mRNA, WT-iPSCs turned out to have comparable levels of SMN to WT fibroblasts, while SMA iPSCs showed significantly reduced levels of SMN full-length transcripts, as a result of the loss of *SMN1* gene. RT-PCR analysis also detected a few truncated transcripts lacking exon 7, along with the full-length SMN transcripts, witnessing the maintenance of functional *SMN2* gene and its alternative splicing. Since lower alpha MNs are the main target of the pathological processes of SMA, iPSCs from SMA and WT fibroblasts were directed towards a motor neuronal and glial fate, in order to disclose in which phase of maturation the pathogenetic events occur. If in the early stages of the differentiation protocol a robust production of MNs was described and no significant difference was documented between the SMA- and WT-derived MNs, interestingly, around week 10 of the protocol, the SMA-derived MNs appeared to be decreased both from a quantitative and a qualitative point of view, as they were fewer and smaller compared to the WT-derived MNs. It was then speculated that the SMA phenotype hampers MN ontogenesis at later developmental time periods, either by inhibiting the generation of new cells or increasing the degeneration of those already matured. This was the first study to report that human iPSCs can be adopted as a reliable model of a genetically inherited disease, such as SMA, and serve as a powerful tool for a better understanding of its etiopathogenesis.

Sareen and Svendsen’s group carried on the experiment for a more precise definition of the mechanisms that are responsible for the post-developmental damage that leads to cell death, as observed *in vitro* [49]. Sareen and colleagues generated two lines of iPSCs from two patients affected by SMA type I and one line of control iPSCs, and then differentiated them into MNs. The first results were consistent with the previous work: after a normal production of MNs at 4 weeks into the differentiation protocol, the cell population underwent a selective reduction in number and size compared to the control iPSC-derived MN cultures. The group ascribed this degeneration to the activation of apoptosis pathways, as suggested by the significantly high percentage of apoptotic cells after 7–10 weeks of differentiation. In particular, the assay of increased levels of apoptotic markers at 8 weeks of differentiation, namely cleaved caspase-3 and caspase-8, supported the assumption that apoptosis plays a crucial role in cell death in SMA iPSCs-derived MN cultures. The intracellular apoptotic signaling cascade is known to be initiated by caspase-8, triggered by the binding of Fas-ligands to their receptors (a TNF family transmembrane protein) [51]. This was confirmed by increased levels of membrane-bound Fas ligand in SMA MNs after 6 weeks of differentiation. The next step was to evaluate whether the inhibition of the Fas-receptor apoptotic pathway could rescue the degeneration of MNs in the SMA lines: the administration of a monoclonal antibody targeting the Fas-receptor (Anti Fas-Ab), starting from the second week of differentiation and for the whole duration of the differentiation process, proved to significantly increase the number of MNs in SMA lines at 8 weeks of differentiation, compared to the untreated cultures [49]. Taken together, these data suggest that an iPSCs-based model of SMA not only may provide new insights on the molecular and pathological processes behind neuronal dysfunction and loss in SMA, but may also represent an invaluable testing ground for the screening and development of new drug compounds, that may eventually be beneficial for patients.

Another contribution to the knowledge on the pathogenic mechanisms of SMA was given by Corti and colleagues [20]. Using a viral- and transgene-free method, they reprogrammed fibroblasts of two type I SMA patients into iPSCs by nucleofecting them with plasmids encoding pluripotency factors (OCT4, SOX2, NANOG, LIN28, c-Myc, and KLF4). A parallel experiment was conducted on a subpopulation of the obtained iPSCs that were treated, and therefore genetically corrected, with *SMN2* sequence-specific oligodeoxynucleotides. The correction of SMA iPSCs consisted in the exchange of a T to C at position +6 of exon 7, thus converting *SMN2* into *SMN1* through the inclusion of exon 7 in SMN2 transcripts. Subsequently, the model required the differentiation of SMA fibroblasts-derived iPSCs, both treated and untreated, into MNs, the cell-type majorly involved in SMA, in order to evaluate the effects of *SMN1* deficiency on these cells. At week 8 of the multistage differentiation protocol, based on the administration of Retinoic Acid and Sonic Hedgehog, they observed specific disease effects of the *SMN1* defect on untreated SMA iPSCs-derived MNs compared to the cultures treated with oligodeoxynucleotides. The statistically significant alterations included an overall reduction in cell soma size, axonal elongation and in the ability to form neuromuscular junctions, resulting in a decreased survival of MNs derived from untreated iPSCs. This experiment well demonstrates that a SMA model reproducing at least some aspects of the disease can be generated, and that MN dysfunction in SMA is the result of multiple neuropathological events likely occurring at a late differentiative state. Genetic correction of *SMN2* via oligodeoxynucleotides proved to rescue the cellular damage to the defective *SMN1* gene and to significantly increase the number of detectable gems, nuclear aggregates of SMN protein which correlate with the rate of translation of the full-length protein.

Given the recent hypothesis on the role of RNA and splicing abnormalities as a primary determinant of selective MN death in SMA [52,53], the experiment was then directed to the analysis of transcriptional changes in untreated SMA iPSC-derived MNs compared to heterozygous iPSC- and treated SMA iPSC-MNs. Gene expression and exon array analysis of RNA revealed a different splicing profile in a subset of genes encoding transcripts that are considered to play a crucial role in SMA pathogenesis, being involved in RNA metabolism, MN differentiation, axonal guidance and signal transduction. Remarkably, the molecular correction with oligodeoxynuleotides proved to shift most of the genes that are differentially expressed or spliced in SMA iPSC-derived MNs towards the heterozygous pattern, further supporting the data on the efficacy of this strategy in correcting the alterations secondary to *SMN1* deficiency.

Following the hypothesis that a cell population other than MNs may be implied in the activation of the apoptotic cascade, the attention was then focused on glial cells. Indeed, astrocytes had already proved to contribute to MN dysfunction in *SOD1* mutated models of ALS [54,55] and to play a key role as mediators of neurodegeneration in a variety of nosological conditions, including Parkinson’s disease [56]. In order to examine morphologic and functional alterations in glial sub-populations, Ebert’s group differentiated human SMA-derived iPSCs and WT iPSCs into astrocytes [50], the most abundant cell type in the central nervous system, that are supposed to be responsible for the maintenance of the perfect environment for neurons’ homeostasis [57]. They described an activated phenotype of SMA astrocytes, as shown by the intense cytoplasmic staining of Glial Fibrillary Protein (GFAP) and Nestin, two components of intermediate filament proteins that are upregulated in reactive glial cells. The activation state was accompanied by significant changes in astrocyte morphology, as they displayed enlarged bodies and thick, short processes. The observation that such alterations occur in the early stages of the differentiation protocol of SMA-derived iPSCs into astrocytes was crucial to infer that the MN loss is preceded by the activation of glial cells, suggesting a causal relationship between the two events. The alterations in the phenotype of astrocytes turned out to be the epiphenomenon of their functional impairments, as ratiometric live-cell calcium imaging reported increased basal calcium levels with a minimal response to ATP in SMA astrocytes compared to WT iPSCs-derived astrocytes. The authors concluded that the defective calcium homeostasis may lead to the activation of apoptotic pathways triggered by the upregulation of pERK1/2 in SMA iPSCs-derived astrocytes, and the resulting increased expression of proinflammatory cytokines like TNF-alpha, IL-1 and IL-6. The ERK programmed cell death cascade was speculated to be initiated also by the deficiency of trophic factors, as witnessed by a decrease in the secretion of Growth Derived Neurotrophic Factor (GDNF), and the whole apoptotic process was attributed to a Fas-mediated mechanism.

Overall, the possibility to isolate cell types that are specifically damaged in the disease process, like MNs and glia cells in SMA, makes iPSCs an unprecedented tool to disclose pathogenetic mechanisms that have been inaccessible so far (Table 1). Moreover, the establishment of reliable disease models is a fundamental milestone in the process of testing novel therapeutic strategies and finally finding a definitive treatment for SMA [58].

## 4. Discussion

The pathogenesis of several MNDs is still obscure under multiple aspects, thus hampering the development of potential therapies [2]. Experimental studies conducted on animal models are crucial, but unfortunately results so far are insufficient [2]. Concerning ALS research, it has been found the overexpression of *SOD1* which characterizes mutant *SOD1* mice may influence observed molecular phenotypes [59]. With regard to SMA, mice physiologically lack the homologous *SMN2* gene, which plays a crucial role in determining clinical phenotypes [60]. As a consequence, data derived from pathogenetic studies need to be validated in human cellular models. Human ESCs have been largely employed for this purpose [37,61], but their use may be limited by ethical issues. Moreover, ESCs are not always available in substantial amount for large scale *in vitro* studies. On the other hand, the development of iPSC-based technology has allowed to generate human pluripotent cells in abundance to perform any *in vitro* study bypassing ethical constraints [22]. Furthermore, a great advantage of iPSCs is that they carry the genetic combination of an individual, thus permitting the study of patient-specific mutations. Issues concerning the heterogeneity of iPSC lines, which could provide misleading results, may be overcome by a careful experimental setting. This should be based on rigorous statistical analysis and standardized protocols. Moreover, the use of cutting-edge molecular methods allows correcting the mutation in patient-derived iPSCs, thus obtaining isogenic controls [44]. Another critical issue is represented by reprogramming techniques: reliable results could be obtained with the use of non-integrative methods or at least sparing the original cell genotype [20]. It is crucial to pay attention to the cellular subtypes obtained in culture with varying differentiation protocols in order to avoid misleading results: well established cellular markers need to be used to assess both the neuronal phenotype and the maturation state. In addition, several studies have highlighted the possibility that reprogrammed cells might maintain an epigenetic memory of the cell of origin, which could interfere with the expression of membrane markers [62]. Recently, advances in reprogramming methods have led to the possibility of directly differentiating mature cells (*i.e.*, fibroblasts or astrocytes) into relevant cell types (*i.e.*, induced neurons) [63]. These rather novel methods are quite efficient in reducing time in culture and speed up the differentiation protocol, but need further assessment. It also needs to be pointed out that pathogenesis studies benefit from long-term observation, from the cell pluripotent state to the mature phenotype, in order to speculate on the time-dependent disease alteration.

## 5. Conclusions

The development and optimization of iPSC-based platforms has allowed elucidating disease-specific mechanisms, which are exquisitely human (Figure 1). Further advances may finally open up the path to a full understanding of key pathogenetic events, leading to the development of effective treatments for ALS, SMA and other MNDs.
